# Efficacy of myofascial induction compared with its simulation on joint amplitude in people with axial spondylarthritis: Protocol of a randomized controlled clinical trial

**DOI:** 10.1371/journal.pone.0286885

**Published:** 2023-10-05

**Authors:** María Alejandra Sánchez Vera, Diego Alejandro Jaimes Fernández, Robert Schleip

**Affiliations:** 1 Faculty of Nursing and Rehabilitation, Physical Therapy Program, Universidad de La Sabana, Chía, Colombia; 2 School of Medicine, Universidad de La Sabana, Chía, Colombia; 3 Department of Sport and Health Sciences, Technical University of Munich, Munich, Germany; Pozan University of Physical Education, POLAND

## Abstract

**Background:**

Axial spondyloarthritis (AxSpA) produces structural changes that cause alterations in body functions. One tissue that seems to have a predictive role in the etiology and progression of the disease is the soft tissue, particularly the fascia. However, little is known about the use of myofascial induction in people with AxSpA, and clinical evidence from physiotherapy regarding potential strategies is limited.

**Objective:**

To evaluate the efficacy of myofascial induction compared with its simulation on joint amplitude in people with AxSpA.

**Methods:**

In this randomized controlled parallel superiority clinical trial, 84 people with an AxSpA diagnosis confirmed by a rheumatologist will be randomly assigned to groups: the experimental group or the control group. The experimental group will receive myofascial induction, and the control group will undergo a simulation of the technique. Both groups will receive an examination session and six intervention sessions twice per week for three weeks. A baseline follow-up will be performed immediately after the intervention and four weeks after treatment.

**Conclusion:**

The results of this study may contribute to a better understanding of the efficacy of myofascial induction for joint mobility in people with AxSpA. The implications of these results have a potential transformative effect on the understanding, analysis, evaluation, and physiotherapeutic treatment of this health condition.

**Trial registration:**

ClinicalTrials.gov NCT04424589. Registered 11 June 2020.

## Introduction

Axial spondyloarthritis (AxSpA) is a chronic and disabling disease that mainly affects young people and causes clear mobility and functional capacity limitations [1]. According to Londoño et al., in Colombia, its prevalence is 0.11% among the population over 18 years of age, with a 3:1 male–female ratio [2]. Although AxSpA is not among the most prevalent rheumatic conditions in the country, it is regarded as an inflammatory disease with higher rates of functional limitation and disability for affected people [3]. Swinnen [4] showed that a combination of personal factors such as depression, impotence due to disease progression, and coping capacity explained approximately 24% of the variation in activity limitations in AxSpA in contrast to biological factors, which represented only 10% of the variation [4].

Given the above information, the management of this condition requires solid interdisciplinary actions that meet the needs of individuals with AxSpA [3, 5]. Although pharmacological therapy is the basis of the treatment of this condition [6], nonpharmacological management is an indispensable complement, since it seeks to improve physical and psychological symptoms, readjust the circumstances of patients according to their conditions, and restore bodily movement [5, 7]. Different studies have shown that pharmacological therapy in conjunction with physical rehabilitation is more effective for managing severe symptoms, improving quality of life, fostering independence in activities of daily living, and optimizing anthropometric measurements related to the transverse muscle diameter and fat percentage than biological therapy, which has been demonstrated to not be equally effective when used alone [4, 5, 8–10].

Based on physiotherapy, various effective strategies have been developed for the management and control of AxSpA [11, 12]. However, orthopedic manual therapy (OMT) techniques focusing on soft tissue management have not been fully studied; therefore, their efficacy is uncertain in this population [13, 14]. Fascia, which is considered a predictor of joint mobility and axial structure and whose basal tone is not dependent on the central nervous system but on the innate resistance of its composition, appears to have a determining role in the etiology of this disease [15, 16].

Masi et al. stated that the increase in this tone at rest can predispose an individual to developing AxSpA [17], because a greater tension load in the fascia limits the transmission of forces from the enthesis to the spine, resulting in inflammatory changes and local mechanical stress [16, 18]. This anomalous tension causes alterations in the composition of the fascial tissue with an increase in myofibroblasts and a decrease in surrounding water, hyaluronic acid, and proteoglycans [17, 19]. These biochemical responses result in an increase in myofascial thickness and superposition of dehydrated collagen layers, triggering clinical pictures consistent with nonspecific and inflammatory pain, functional limitation, and loss of muscle elasticity among people with AxSpA [20, 21]. These signs become the therapeutic targets of myofascial induction, which is understood as a technique involving guided mechanical forces of low load and a long duration. Its effectiveness has been demonstrated in different mechanical and inflammatory pathologies, and its mechanism of action allows reorganization of fibroblasts and collagen layers, contributing to transformation of the fundamental substance from a densified state to a more fluid state, which results in clinically better patterns of movement in patients [10, 13, 22–24].

### Primary objective

The objective of this study will be to evaluate the efficacy of myofascial induction compared to its simulation on joint amplitude in people diagnosed with AxSpA.

### Secondary objective

The secondary objective will be to describe changes in the quality of life and functionality of people with AxSpA between the groups analyzed.

### Hypothesis

The myofascial induction group will have better results than the simulation group with respect to joint mobility among people with AxSpA.

## Materials and methods

### Design

This will be a randomized, superiority, double-blind, parallel clinical trial. The trial was designed according to the recommendations for intervention trials (SPIRIT) [25] and the CONSORT guidelines [26]. This protocol complies with the Helsinki guidelines for research involving humans and has been approved by the research and ethics subcommittee of the Faculty of Nursing and Rehabilitation of the Universidad de La Sabana (021-18-2020.) All participants will receive an informed consent form with all the procedures and the risks and benefits of the study. To protect the identity of the participants, each of them will be assigned a numerical code.

### Study environment

This study will be conducted in an outpatient service center for people with rheumatic diseases in the city of Bogotá, Colombia.

### Inclusion criteria

- People of both sexes over 18 years of age.
- People with a rheumatology-confirmed diagnosis of AxSpA regardless of the level of disease activity.
- People with the cognitive ability to follow orders.
- People who agree to participate in the study.

### Exclusion criteria

- People receiving oral or parenteral coagulation therapy.
- Pregnant women.
- People with kinesiophobia.
- People with previous physical therapy in the last 15 days.
- The presence of active cancer or current treatment with chemo- or radiotherapy.

### Intervention

The study interventions are based on the myofascial induction techniques proposed by Andrezj Pilat [21]. The physiotherapist (PT) who performs this procedure in the experimental group will be trained and certified in this technique by the author in question. All techniques will be performed in all planned treatment sessions. 6 myofascial induction and simulation sessions will be done, two per week, every other day, for 3 weeks. Patients will be followed up one month after the last session with final evaluation.

### Experimental group intervention

#### J stroke

After a palpatory examination of the fascia, with the participant in prone position, the PT will apply slight superficial traction with his nondominant hand, which helps maintain tension on the myofascial tissue of the dorsolumbar spine. With his dominant hand, he will move his third finger up to the second level of the distal phalanx while the forearm is pronated, and then he will perform the “J” stroke, slowly sliding with nonpainful superficial depth, and rapid supination in the shape of a “J” on the site of the myofascial restriction. For each of the sites perceived as adhered or restricted, an average of seven J strokes should be performed. This technique does not require any lubricant or contact medium.

#### Release of the paravertebral fascia

The participant will be in a sitting position with the back uncovered, the contacts of the PT will be the knuckles of the second, third, and fourth fingers, the participant will be asked to flex the head, and the contacts will be moved up to the second thoracic vertebra. Once the movement is stopped, the therapist will bring back one of his lower extremities. At this point, he will continue the maneuver, keeping the elbows in extension and leaning on his body mechanics to gain depth. Then, he will ask the participant to slowly flex forward while keeping the legs in abduction while the PT slides the contacts caudally until reaching the high lumbar vertebrae. At each site where a movement restriction is perceived, he will stop without increasing the pressure for seven seconds. This process should be repeated three times.

#### Technique for releasing the quadratus lumborum

The participant will be in the lateral decubitus position with knee and hip flexion of 90°. The PT will remain bipedal in front of the patient at the height of the torso, the cranial hand will fix the ribs in an ascending direction, and the distal hand will be positioned with the ulnar edge of the hand in the space between the iliac crest and the last rib. The PT will seek to gain depth in this space until resistance is felt. Once he reaches the maximum allowed tissue barrier, he will perform a rhythmic, low-amplitude, flexion and extension movement of the elbow for 15 cycles in three repetitions.

In a following moment, in the same position, the patient will slightly drop the legs outside the table, the PT will accompany this tensioning of the quadratus lumborum, locating the pads of the fingers within the common paravertebral mass, and the PT will perform a slide his fingers in the transverse direction without generating flexion of the carpus. He will perform 13 cycles in three repetitions.

This technique will end with the patient in the lateral decubitus position, the legs will fall slightly outside the table, and the PT will have his hands crossed, with one on the iliac crest and another on the rib cage. He will begin by applying light traction on the skin, and then with light pressure, he will try to overcome the barriers of the tissue by facilitating movement of the fascia with sustained pressure for five minutes.

#### Release of the sacroiliac fascia

The patient will remain prone with knee flexion at 90°, the PT will remain bipedal next to the limb to be worked on, and the PT’s cranial hand will rest on the sacrum in the direction of the iliac crests while maintaining internal rotation of this limb with the caudal hand. He will maintain the pressure until it surpasses between three and six tissue barriers, which should be sustained for four minutes.

#### Sustained pressure technique in the upper trapezius

For the neck, the myofascial release technique will be used in the upper trapezius, the patient will be supine, and the PT will position his hands at the head of the table while seated as follows: His nondominant hand will support the patient. The dominant hand with 90° flexion will be at the level of the metacarpophalangeal joints, and the initial contact with the upper trapezius and angular muscles of the scapula will begin gently and progressively with the tips of the fingers, increasing the pressure and depth of the position of the hands. The PT will feel each of the tissue barriers and slowly yield to the pressure. This position will be maintained for five minutes.

#### Cross-hand technique in thoracolumbar fascia

The intervention continues with the cross-hand technique; at this time, the PT will be located on one side of the table, and the patient will have the back uncovered and be in the prone position. The PT will synchronize his breathing with that of the patient. Once he has taken two deep inhalations, the therapist will place the palms of his hands completely on the patient’s back at the level of the lumbosacral region with the fingers in slight abduction, and he will begin applying light traction on the skin, bringing the tissue to a state of pretension; slight pressure will be maintained for five minutes. Once the technique is complete, the pressure will be slowly released, and before removing the hands from the segment, manual contact will be maintained for 10 seconds. Once the hands are removed, the patient will be allowed to rest on the table for approximately two minutes to avoid vasovagal responses.

#### Control group intervention

The simulation intervention in the control group will suggest modification of basic parameters that does not alter the visual demonstration of the technique. The PT assigned to this group will omit the basic principles of myofascial induction: traction, sustained pressure, and tissue depth. All the proposed strategies for the experimental group will be carried out.

#### Discontinuation of participation

During the course of the study, any participant can choose to withdraw from the trial at any time. Participants can choose to suspend treatment and/or evaluations in the study but can remain in the follow-up if they wish; however, participants can also withdraw their consent, which implies that they wish to completely withdraw from the study, completely restricting the researchers’ access to the data obtained to date and those participants’ clinical histories.

### Outcomes

**Fig 1:**
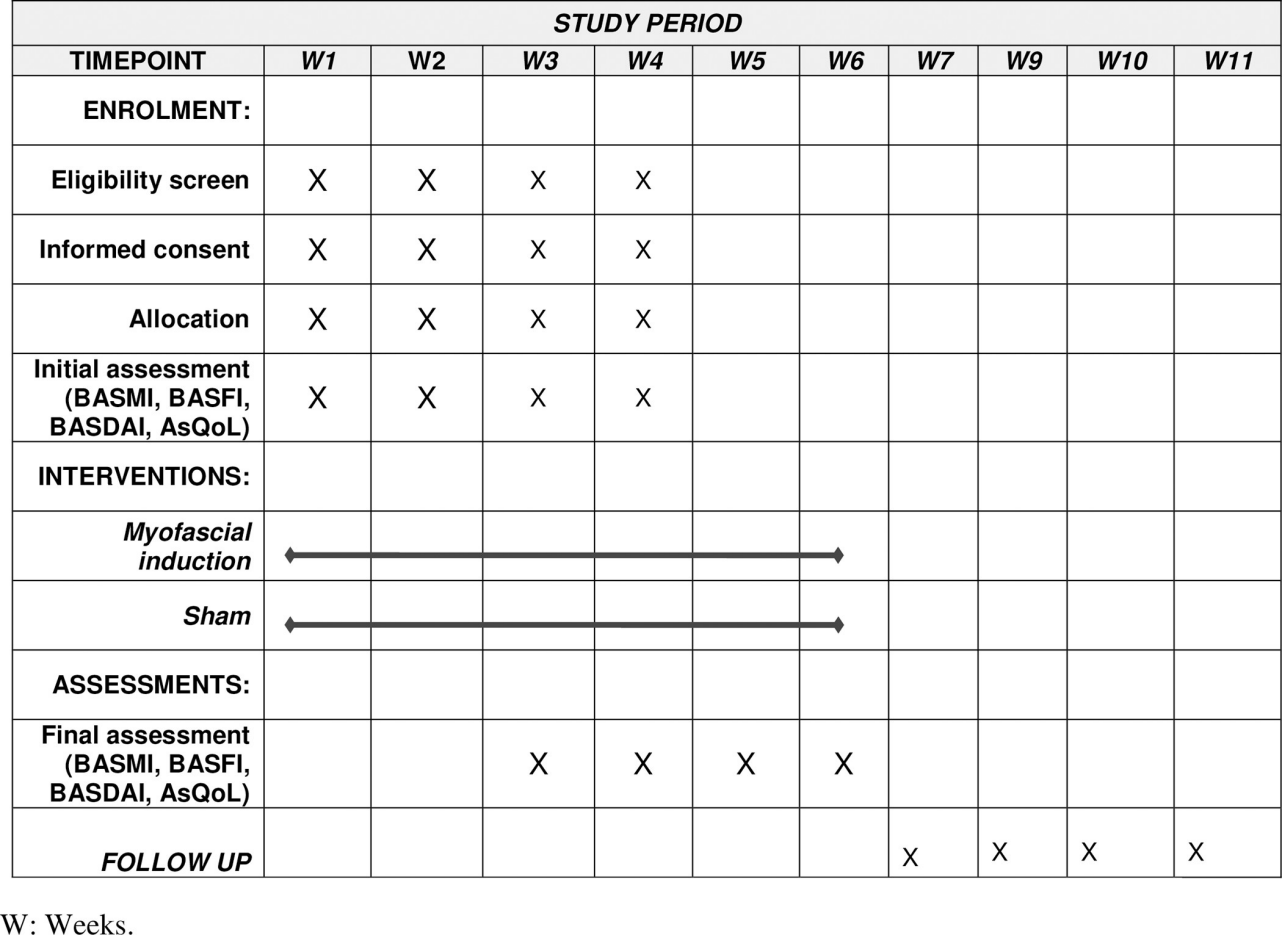
Standard Protocol Items: Recommendations for Interventional Trials (SPIRIT) timeline of measurements. Time represented by weeks (WK).

**Fig 2:**
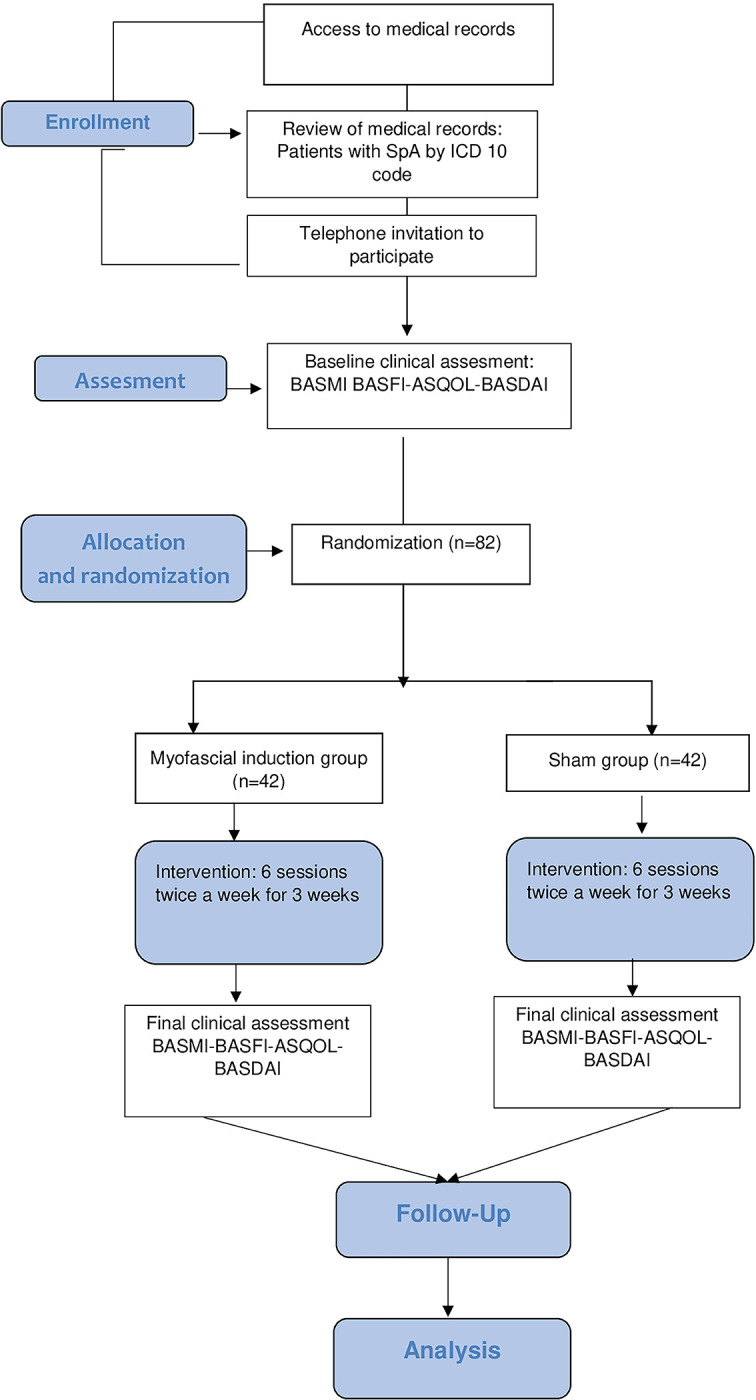
Study flow diagram, adapted from CONSORT.

The primary outcome will be joint mobility measured by the Bath Ankylosing Spondylitis Metrology Index (BASMI). The secondary outcomes will be the Bath Ankylosing Spondylitis Functional Index (BASFI), Bath Ankylosing Spondylitis Disease Activity Index (BASDAI), and the Ankylosing Spondylitis Quality of Life Questionnaire (AsQol) [27, 28]. Each of these variables will be measured before the intervention, after the intervention, and four weeks after the intervention. The participants will undergo an initial evaluation before randomization. The results will be collected at each time point according to (Figs 1 and 2).

### BASMI

The index that groups four axial measurements (cervical rotation, Schober test, lateral torso flexion, tragus-to-wall distance) also includes a measurement of peripheral mobility (intermalleolar distance) [29]. These variables will be measured comparatively at least twice to obtain the mean measurements to calculate the final score based on a table of 11 points [25]. This index is evaluated by the examiner and conveys spinal and peripheral mobility findings in centimeters and degrees [27, 30].

### BASFI

The BASFI questionnaire is self-administered and consists of 10 questions investigating the functional status of the person diagnosed with AxSpA. Each question can be scored from 0 to 10, with 0 corresponding to no limitation in carrying out the proposed activity and 10 corresponding to the absolute impossibility of carrying it out. This scoring system employs a visual analog scale scored on a horizontal metric line [31]. The final score is obtained from the sum of the results of the questions divided by 10. This instrument is validated for use in Spanish in Colombia [32, 33].

### BASDAI

The BASDAI evaluation is self-administered and consists of six questions inquiring about issues related to the five main symptoms of AxSpA: fatigue, axial pain, joint pain and/or edema, enthesis pain, and morning stiffness [34]. Similar to the BASFI, it is based on a visual analog scale measuring 10 centimeters in a horizontal line. It is scored by adding the scores for questions 1 to 4; for questions 5 and 6, the mean is calculated, and finally, the scores of all questions are added and divided by 5. A higher BASDAI score suggests more active disease [27]. This questionnaire is also validated in Spanish [34].

### AsQoL

The AsQoL is self-administered and validated in Spanish in Colombia. It consists of 18 questions with the possibility of dichotomous answers (YES—NO). The affirmative answers are scored as 1, and the negative answers are scored as 0. The overall score is the sum of the scores for each question and can be between 0 (better health-related quality of life) and 18 (worse health-related quality of life). If more than three questions are unanswered, the overall score cannot be calculated [35].

### Recruitment

The people who will be eligible for the study are those receiving some medical or therapeutic service in the clinic selected for the study. A research assistant will review the medical records of people with an AxSpA diagnosis confirmed by a rheumatologist. Individuals who are potentially eligible will be contacted by telephone, and the intention and scope of the study will be briefly explained. After this communication, if a person verbally expresses their desire to be part of the research process, a face-to-face interview will be scheduled.

### Sample size

The sample size was calculated in the program R Studio version 3.5.3, package of superiority clinical trials, based on a mean difference of 4.0 in the BASMI with a standard deviation of 2, an r (ratio) between groups of 1, and assuming a loss of 20%. An alpha one sided of 0.05% and a power of 0.80 were estimated based on mean difference test corresponding to a clinical trial of superiority (repeated measures ANOVA). The total number of people was 82 (42 per arm). Non probabilistic purposive sampling will be used.

### Randomization and concealment

In this study, a simple randomization process will be used by assigning random numbers without repetition in Excel. For randomization concealment, opaque, sealed, consecutively numbered envelopes will be used and kept by a single person. An external person will prepare the envelopes. An envelope will be opened in sequence once the participant has completed the baseline physical examination and the corresponding measurements.

### Blinding

The people who will perform the baseline and final evaluations will not know the randomization sequences, nor will they be involved in the execution of the treatments in the control group or the experimental group. The participants will visually receive the same procedures since the techniques for the control and experimental groups appear the same; accordingly, they will also be blinded. The PTs who will perform the interventions cannot be blinded due to the need to know the principles of the technique and the form of execution to guarantee correct application of the treatment or simulation. The data analysis will be performed by an external evaluator in the intervention and evaluation process.

### Data management

The data obtained from the participants will be recorded in virtual record format on the REDCap platform [36] by means of a double digitization process to avoid errors in data recording. This platform requires a username and password that will be known only to the research coordinator. In the case of any eventuality, the primary source will be contacted, and the value will be corrected. All documents will be safely stored confidentially. Participants will be identified by a specific number or unique test code. The name and any other identifying details will not be included in any part of the database.

### Statistical analysis

The data will be analyzed in the latest version of the R Studio program. A statistical analysis will be performed based on the purposive analysis principles. The assumption of normality for the quantitative variables will be verified with the Shapiro–Wilk test, followed by a descriptive analysis; for the continuous variables, measures of central tendency or dispersion according to the data will be applied. For categorical variables, the proportions and frequencies will be calculated. Differences between the groups in measurements for the primary and secondary objectives will be analyzed by repeated measures ANOVA the variables with a normal distribution. Nonparametric tests will be performed if the data do not comply with the assumption of normality, interaction of arm by time effect will not be tested. Qualitative variables will be evaluated with the chi-square test of independence or Fisher’s exact test, as appropriate. For all tests, a significance level of p <0.05 will be employed.

### Damage

All adverse events that are found during the execution of the clinical trial should be reported in a timely manner, all the data will be collected according to Common Terminology Criteria for Adverse Events (CTCAE) version 5.0. This information will be recorded in the report format that includes description of the event, start date and end date, relationship with physiotherapeutic treatment and measures taken on the event. Follow-up information will be provided as needed. Each of these formats will be recorded with the serial code assigned to each patient [29, 37–39].

### Monitoring

Strict compliance with the protocol and accuracy in relation to the source documents will be evaluated. Following the standard operating procedures declared in this document, an external monitor or referee who is not involved in the research will randomly verify that the clinical trial is carried out within the approved and established parameters and that the data generated are documented and reported in accordance with the protocol every two weeks.

## Discussion

Physiotherapeutic intervention is undoubtedly the first-choice treatment for the management of patients with AxSpA; however, studies based on manual therapy with an emphasis on soft tissue are still insufficient [7, 9, 40]. The results of this research can contribute to the understanding and formulation of new treatment paradigms with rapid therapeutic action that complement the strategies that have been shown to be more effective, such as aerobic exercise and resistance and neuromuscular training [1, 5, 7, 41]. Likewise, it will clarify the role of soft tissue in joint mobility in patients with AxSpA [5, 19].

The results of this research seek to initially understand the influence of the fascia on the spinal mobility of patients with this health condition. Fascial hypertonicity, as proposed by Masi et al. [18] may be a "missing link" in therapeutic understanding of AxSpA. The modulation of fascial and soft tissue tone can become a treatment target from the physiotherapist’s actions to achieve hard declines such as function, quality of life and pain.

This study is formulated with high methodological quality, allowing its reproducibility in any clinical scenario. The detailed description of the intervention strategies allows any PT to accurately perform a myofascial induction intervention in this type of patient. Additionally, to date, this is the first study focusing exclusively on evaluating the efficacy of manual therapy techniques, particularly myofascial induction, in patients with AxSpA. The outcomes in this study are intended to elucidate changes in mobility and function; however, these can lead to more research questions with a clinical, biochemical, or biomechanical focus, contributing to the formulation of new disciplinary approaches. Unfortunately, for this study, blinding of the therapists performing the sessions will not be possible, and given the strict eligibility conditions proposed, the results will not be generalizable to another type of population, even a population with a similar rheumatic disease.

## Supporting information

S1 Checklist SPIRIT 2013 checklist: Recommended items to address in a clinical trial protocol and related documents*. (DOCX)

S1 File Protocol. (DOCX)

S2 File (DOCX)
