# Supplementary material for: Efficacy of myofascial induction compared with its simulation on joint amplitude in people with axial spondylarthritis: Protocol of a randomized controlled clinical trial
Source: PLoS One. 2023 Oct 5;18(10):e0286885. doi: 10.1371/journal.pone.0286885 (PMC10553292; doi:10.1371/journal.pone.0286885)
Supplement: S1 File — (DOCX) [file pone.0286885.s002.docx]

**EFFICACY OF MYOFASCIAL INDUCTION COMPARED WITH ITS SIMULATION ON JOINT AMPLITUDE IN PEOPLE WITH AXIAL SPONDYLARTHRITIS: PROTOCOL OF A RANDOMIZED CONTROLLED CLINICAL TRIAL**

# PRINCIPAL INVESTIGATOR CONTACT

| **Principal investigator** | María Alejandra Sánchez Vera, Physiotherapist, Orthopedic Manual Therapy Specialist, Master of Science in Epidemiology. Universidad de La Sabana. Chía, Colombia. |
| --- | --- |
| **Sponsor** | Universidad de La Sabana. |
| **Funders** | N.A |
| **Committee** | N.A |

# SUMMARY

| **TITTLE** | Efficacy of myofascial induction compared with its simulation on joint amplitude in people with axial spondylarthritis: protocol of a randomized controlled clinical trial |
| --- | --- |
| **Clinical trials register** | NCT04424589 |
| **Funding** | Universidad de La Sabana |
| **Desing** | Parallel randomized clinical trial of superiority, double blind, controlled. |
| **Trial participants** | Adults diagnosed with axial spondyloarthritis who attend the rheumatology service of an outpatient clinic in the city of Bogotá. |
| **Sample size** | 82 people. |
| **Planned trial period** | The intervention per participant is designed to develop 6 therapy sessions twice a week over the course of 3 weeks. A follow-up will be carried out at the fourth week post-intervention where the same baseline evaluation measures will be collected. |

**3. OBJECTIVES AND OUTCOME MEASURES**

| **OBJECTIVES** | **OUTCOME MEASURES** | **MEASUREMENT TIME** |
| --- | --- | --- |
| **General objective:**  **T**o evaluate the efficacy of myofascial induction compared with sham manual therapy on joint range on BASMI change in patients diagnosed with axial spondyloarthritis. | **BASMI:** The global score of this index will be analyzed, as well as the mean difference of each of the variables that make it up: (Intermalleolar distance, tragus wall distance, Schober test, cervical rotation, lateral flexion test). | At week 1 at the exam session, the basal measurement is carried out among the participants, at week 3 session 6 the final measurement is carried out, at the fourth week post-intervention the variables will be measured again. |
| To describe changes in terms of quality of life, and functionality of the AxSpace between the groups through the analysis of the scores of the BASFI and ASQoL indices. | BASFI: Allows the measurement of the level of functionality of patients with EspaAx ASQoL: Allows the measurement of the level of quality of life among patients with AxSpA. | At week 1 at the exam session, the basal measurement is carried out among the participants, at week 3 session 6 the final measurement is carried out, at the fourth week post-intervention the variables will be measured again. |

**PARTICIPANT IDENTIFICATION**

**Trial participants**

Participants of legal age with a diagnosis of axial spondyloarthritis who attend an outpatient clinic in Bogotá will be included. This diagnosis must be confirmed by a rheumatologist and must be recorded in the clinical history. Patients will be included regardless of the type of AxSpa, the level of activity, severity, or time of diagnosis of the disease.

**Inclusion criteria**

- Patients of both sexes over 18 years of age.
- Patients with rheumatology-confirmed diagnosis of AxSpace regardless of the level of disease activity.
- Patients with cognitive ability to follow orders.
- That they agree to participate in the study and sign informed consent.

**Exclusion criteria:**

- Patients receiving oral or parenteral coagulation therapy.
- Pregnant women
- Patients with Kinesiophobia.
- Patients with previous physiotherapy in the last 15 days.
- Presence of active cancer, current chemotherapy, or radiotherapy treatment.
- Patients who do not wish to participate in the study.

**RECRUITMENT:**

A research assistant will review the medical records of patients who have a diagnosis of AxSpa confirmed by a rheumatologist. It is to be expected that in this first screening some records do not meet the inclusion criteria; however, for those patients who are potentially eligible, the research assistant will contact them by phone and briefly explain the intention and scope of the study. After his communication, if the patient verbally expresses his desire to be part of the research process, a face-to-face meeting space will be scheduled to explain the dynamics of the study.

Once the face-to-face meeting with the possible participant is carried out, in this session the informed consent will be sufficiently and completely explained to them, stating verbally and in writing the potential risks and advantages of this therapeutic process, likewise carried out the baseline examination process with the indices of functionality, mobility, disease activity and quality of life related within the investigation. Based on this evaluation, patients who meet the inclusion criteria will be considered.

Following due process, the patient will be randomized into one of the groups (intervention or control) and will attend 6 sessions of myofascial induction or simulated manual therapy according to the assigned group, in the last session the final assessment will be made with the same indices that were initially assessed. Patients who received sham manual therapy will receive the real MFI after completion of the RCT, if they so choose.

**RANDOMIZATION AND CONCEALMENT**

In this study, a simple randomization process will be used by means of the assignment of random numbers without repetition carried out by means of the Excel program. This process will be carried out by an external person unrelated to the investigation and methodological rigor will be guaranteed by means of an additional external witness or arbitrator. This process will be carried out once the patient has had the examination session and, as mentioned above, it will be carried out by a person unrelated to the investigation. To conceal randomization, consecutively numbered, sealed, opaque envelopes kept by a single person will be used and maintained. An external person will prepare the envelopes. An envelope will be opened in sequence once the participant has completed the baseline physical examination and corresponding measurements. The allocation will be informed to the treating physiotherapist by email before the participant attends the for treatment.

**Blinding**

To guarantee double blinding, the people who will carry out the baseline and final evaluations will not know the randomization sequences, they will not be involved in the execution of the treatments of the control group or the experimental group, the patients will receive the same procedures visually since the simulated techniques look exactly equal, in this way they will also be armored. The physiotherapists who will carry out the interventions cannot be masked due to the need to know the principles of the technique and its form of execution to guarantee a correct application of the treatment and the respective simulation.

**PROCEDURES**

1. Reading medical records to filter patients potentially candidates for the study.
2. Make an invitation to participate by phone, if the response is positive, schedule a face-to-face meeting.
3. In the face-to-face meeting, a researcher who does not participate in the interpretation and analysis of the data must carry out the process of detailed reading of the informed consent. Once accepted, the baseline examination will proceed if the patient meets the selection criteria, through the physical data collection format, applying the indices: BASMI, BASDAI, BASFI, ASQoL. The form of application of these indices will be carried out as follows:

**BASMI** (1,2)**:**

| **EXTENT** | **INITIAL POSITION** | **METHOD** | **RECOMMENDATIONS** |
| --- | --- | --- | --- |
| Tragus wall distance | The patient stands barefoot, with his back against the wall, shoulders ideally level, shoulder blades, buttocks, and heels against the wall. Feet must remain parallel. The examiner should ensure alignment of the head in the most neutral position (anatomical alignment) possible. | The patient stretches the chin as much as possible (retraction). The examiner observes carefully and stands next to the patient on the wall to measure the distance between the ear tragus and the wall, using a rigid ruler. This procedure is carried out twice, choosing the best of the measurements, the average of both sides allows obtaining the result | The examiner must ensure that cervical extension, rotation, flexion, or lateral flexion does not occur. Prevent the chin from exceeding the horizontal. In addition, the patient maintains the retraction while the right and left measurement is performed. |
| trunk lateral flexion | The patient stands barefoot, with his back against the wall, shoulders ideally level, shoulder blades, buttocks, and heels against the wall. The feet must remain parallel and separated on their outer edges at approximately 30 cm. | Before any movement occurs, the patient should keep the arms, wrists, and fingers close to the body, with the fingers extended and the shoulders depressed.  The examiner should measure from the tip of the middle finger to the floor. With arms relaxed at the sides, the patient will attempt to reach the floor by lateral bending and maintaining shoulder depression. The examiner should again measure from the tip of the middle finger to the floor.  The difference between 2 measurements represents the amount of lateral flexion. Repeat in the other side. The result is the average of the results on both sides and the best of the attempts is recorded | The examiner should ensure that the patient keeps the arms, fingers, and knees straight and the heels on the floor. Also make sure to avoid any forward flexion, extension or rotation of the trunk. |
| Lumbar flexion (modified Schober) | Bipedal patient with feet parallel at 30cms measured from the outer edges. The examiner marks a first point on the fifth lumbar vertebra L5 (50) midway to the posterosuperior iliac spines, a second point is marked 10 cm above this and a third point 5 cm below the first to give a line overall 15 cm. | The patient should perform trunk forward flexion with the knees fully extended. The distance between the 2 upper and lower marks is measured with a tape measure. Any increase beyond 15 cm represents the amount of movement achieved. Make 2 attempts and take the average of the measurements. | At the end of the movement, the examiner might choose to allow a slight knee flexion to lessen the influence of the hamstrings. This must be documented in the initial data recording form. |
| cervical rotation | Patient in a seated positiON, feet resting on the floor, maintaining 90° knee flexion, the fulcrum of the goniometer is in the center of the patient's head, the mobile arm will follow the nose and the fixed arm the acromion of the side towards which rotation occurs. | The patient performs an active cervical rotation to the right and to the left. The procedure is done twice for both sides and the best of the attempts is recorded, the average of both is the result. | The examiner must ensure that the patient is not neck flexing or lateral flexing. |
| Intermalleolar distance | The patient is in a supine position with the knees extended. | The examiner proceeds to separate the patient's feet as far as possible by abducting the hips bilaterally. The distance between the medial malleoli is measured with a tape measure. | To avoid this being a painful measurement for the patient, perform a quick measurement. |

At the end, you must record the score given by the patient for each of the variables, as well as the total score for the analysis between groups. The score will be made using the 11-point BASMI SCALE, the researcher should continue with the assessment of the BASFI, BASDAI, ASQOL, BASMI.(3)

**Intervention****:**

The study interventions are based on the myofascial induction techniques proposed by Andrezj Pilat The physiotherapist (PT) (4) who performs this procedure in the experimental group will be trained and certified in this technique by the author in question. All techniques will be performed in all planned treatment sessions. 6 myofascial induction and simulation sessions will be done, two per week, every other day, for 3 weeks. Patients will be followed up one month after the last session with final evaluation.

**Experimental group intervention**

**J stroke:**

After a palpatory examination of the fascia, with the participant in prone position, the PT will apply slight superficial traction with his nondominant hand, which helps maintain tension on the myofascial tissue of the dorsolumbar spine. With his dominant hand, he will move his third finger up to the second level of the distal phalanx while the forearm is pronated, and then he will perform the “J” stroke, slowly sliding with nonpainful superficial depth, and rapid supination in the shape of a “J” on the site of the myofascial restriction. For each of the sites perceived as adhered or restricted, an average of seven J strokes should be performed. This technique does not require any lubricant or contact medium

**Release of the paravertebral fascia:**

The participant will be in a sitting position with the back uncovered, the contacts of the PT will be the knuckles of the second, third, and fourth fingers, the participant will be asked to flex the head, and the contacts will be moved up to the second thoracic vertebra. Once the movement is stopped, the therapist will bring back one of his lower extremities. At this point, he will continue the maneuver, keeping the elbows in extension and leaning on his body mechanics to gain depth. Then, he will ask the participant to slowly flex forward while keeping the legs in abduction while the PT slides the contacts caudally until reaching the high lumbar vertebrae. At each site where a movement restriction is perceived, he will stop without increasing the pressure for seven seconds. This process should be repeated three times.

**Technique for releasing the quadratus lumborum:**

The participant will be in the lateral decubitus position with knee and hip flexion of 90°. The PT will remain bipedal in front of the patient at the height of the torso, the cranial hand will fix the ribs in an ascending direction, and the distal hand will be positioned with the ulnar edge of the hand in the space between the iliac crest and the last rib. The PT will seek to gain depth in this space until resistance is felt. Once he reaches the maximum allowed tissue barrier, he will perform a rhythmic, low-amplitude, flexion and extension movement of the elbow for 15 cycles in three repetitions.

In a following moment, in the same position, the patient will slightly drop the legs outside the table, the PT will accompany this tensioning of the quadratus lumborum, locating the pads of the fingers within the common paravertebral mass, and the PT will perform a slide his fingers in the transverse direction without generating flexion of the carpus. He will perform 13 cycles in three repetitions.

This technique will end with the patient in the lateral decubitus position, the legs will fall slightly outside the table, and the PT will have his hands crossed, with one on the iliac crest and another on the rib cage. He will begin by applying light traction on the skin, and then with light pressure, he will try to overcome the barriers of the tissue by facilitating movement of the fascia with sustained pressure for five minutes.

**Release of the sacroiliac fascia:**

The patient will remain prone with knee flexion at 90°, the PT will remain bipedal next to the limb to be worked on, and the PT’s cranial hand will rest on the sacrum in the direction of the iliac crests while maintaining internal rotation of this limb with the caudal hand. He will maintain the pressure until it surpasses between three and six tissue barriers, which should be sustained for four minutes.

**Sustained pressure technique in the upper trapezius:**

For the neck, the myofascial release technique will be used in the upper trapezius, the patient will be supine, and the PT will position his hands at the head of the table while seated as follows: His nondominant hand will support the patient. The dominant hand with 90° flexion will be at the level of the metacarpophalangeal joints, and the initial contact with the upper trapezius and angular muscles of the scapula will begin gently and progressively with the tips of the fingers, increasing the pressure and depth of the position of the hands. The PT will feel each of the tissue barriers and slowly yield to the pressure. This position will be maintained for five minutes.

**Cross-hand technique in thoracolumbar fascia:**

The intervention continues with the cross-hand technique; at this time, the PT will be located on one side of the table, and the patient will have the back uncovered and be in the prone position. The PT will synchronize his breathing with that of the patient. Once he has taken two deep inhalations, the therapist will place the palms of his hands completely on the patient’s back at the level of the lumbosacral region with the fingers in slight abduction, and he will begin applying light traction on the skin, bringing the tissue to a state of pretension; slight pressure will be maintained for five minutes. Once the technique is complete, the pressure will be slowly released, and before removing the hands from the segment, manual contact will be maintained for 10 seconds. Once the hands are removed, the patient will be allowed to rest on the table for approximately two minutes to avoid vasovagal responses.

**Control group intervention:**

The simulation intervention in the control group will suggest modification of basic parameters that does not alter the visual demonstration of the technique. The physical therapist assigned to this group will omit the basic principles of myofascial induction: traction, sustained pressure, and tissue depth. All the proposed strategies for the experimental group will be carried out.

**ADVERSE EVENTS**

Adverse events will be evaluated in the format designated for this purpose. In general terms, the report format includes information that will be completed by the patient and other information that must be organized by the principal investigator. included adverse events will be those that occurred during or within 24 hours of treatment. The adverse event questionnaire generally contains questions related to whether the patient had experienced this sensation before during the intervention procedures, likewise it focuses on finding out how many hours the event lasted and to what extent the adverse event would have caused physical discomfort in the patient. This will be measured through a numerical rating such as the 11-point Visual Analogue Scale (VAS) from 0 to 10 (0 = it doesn't bother you at all and 10 = it bothered you in the worst possible way).(5)

Whether or not an adverse event is of sufficient severity to require withdrawal of the participant's treatment will be left to the investigator's clinical judgment. A participant may also voluntarily withdraw from treatment due to what he or she perceives to be an intolerable adverse event. If any of these occur, the participant should undergo an end-of-trial assessment and receive appropriate care under medical or physiotherapy supervision until symptoms resolve or condition is stable.

**DATA MANAGEMENT:**

The data obtained from the participants will be recorded in virtual record format on the REDCap platformby means of a double digitization process to avoid errors in data recording. This platform requires a username and password that will be known only to the research coordinator. In the case of any eventuality, the primary source will be contacted, and the value will be corrected. All documents will be safely stored confidentially. Participants will be identified by a specific number or unique test code. The name and any other identifying details will not be included in any part of the database.

**SAMPLE SIZE:**

To calculate the sample size of the trial, the article by Silte et al (6)is taken as a reference, since there are no cut-off points in Colombia or clearly established in Latin America that differentiate BASMI normality scores between the population with and without the presence of AxSpa. Furthermore, to date there is no trial available in this population using myofascial induction as the primary intervention or experimental techniques. Given the above, it is necessary to cite a theoretical reference to make the estimates of the sample size calculation. A study was chosen that complied with the analysis of each of the BASMI variables and that developed a comparison between groups of two techniques and strategies from physiotherapy.

Based on the foregoing, the study in question is taken as a reference, taking the means of the control and experimental group post-intervention with their respective standard deviations for each of the variables of the BASMI index, followed by this, it is estimated and constructed among the researchers of the study of the expected deltas that are considered relevant in routine clinical practice in physiotherapy and rheumatology clinics.The calculation is carried out using the latest version 3.5.3 of the R Studio program, downloading the EpiR library, together with the superiority clinical trials package called Epi.supc, once this process is finished, the following code is run:

epi.supc(treatment=1, control=1, sd=1, delta=1, n=NA, r=1, power=0.80, alpha=0.05)

Being treat the expected average in the experimental group, control the expected average in the control group, sd the standard deviation, delta the minimum change that represents clinical importance, r the ratio between groups. An alpha of 0.05% and a power of 0.80 were estimated for all calculations. This calculation was made for each of the BASMI variables (trunk anterior flexion, trunk lateral flexion, cervical rotation, intermalleolar distance, tragus wall distance) and the largest calculated sample size was selected, which was 33 patients per arm. Considering a percentage of loss of participants of 20%, the total number of patients is 82 in total (42 per arm). A non-probabilistic sample was ensured by intention.

**STATISTICAL ANALYSIS:**

The data will be analyzed in the latest version of the R Studio program. A statistical analysis will be performed based on the purposive analysis principles. The assumption of normality for the quantitative variables will be verified with the Shapiro–Wilk test, followed by a descriptive analysis; for the continuous variables, measures of central tendency or dispersion according to the data will be applied. For categorical variables, the proportions and frequencies will be calculated. Differences between the groups in measurements for the primary and secondary objectives will be analyzed by an analysis of variance (ANOVA) for the variables with a normal distribution. Nonparametric tests will be performed if the data do not comply with the assumption of normality. Qualitative variables will be evaluated with the chi-square test of independence or Fisher's exact test, as appropriate. For all tests, a significance level of p <0.05 will be employed.

**ETHICS PROCEDURES**

From its approach and formulation, this clinical trial adheres to national and international ethical regulation standards, particularly the Declaration of Helsinki promulgated by the World Medical Association as a proposal for ethical principles for medical research in humans. This study seeks to carry out and evaluate the efficacy of a procedure that has potential therapeutic value that does not affect or endanger the physical integrity or health of the participants. As this statement cites, the importance of the objective pursued in the study is greater than the risk and costs for the participant. Likewise, it is reiterated that the participation of the person in this clinical trial is completely voluntary and regulated through informed consent that must be approved by an ethics committee as well as the research protocol. It is important to mention that in accordance with the declaration, the privacy and intimacy of the participants will be permanently guaranteed with respect to the registration of the data and the disclosure of these.(7)

**REFERENCES**

1. Thomas A, Martindale J. An updated synopsis of the Bath Indices – outcome measures for use with Ankylosing Spondylitis patients and their broader application. :16.

2. Castro Villegas M del C, Batlle Gualda E. Metrología en espondiloartritis. Reumatol Clin. 1 de marzo de 2010;6:11-7.

3. Calin A, Jones SD, Garrett SL, Kennedy LG. Bath Ankylosing Spondylitis Functional Index. Br J Rheumatol. agosto de 1995;34(8):793-4.

4. Pilat A, Gonzalez Nieto JL, Batuecas Suárez A. Terapias miofaciales : inducción miofascial. Aséctos teóricos y aplicaciones clínicas / Andrzej Pilat ; revisión técnica Alicia Batuecas Suárez, José Luis González Nieto. [Internet]. 2003 [citado 27 de marzo de 2019].

5. Carnes D, Mars TS, Mullinger B, Froud R, Underwood M. Adverse events and manual therapy: a systematic review. Man Ther. agosto de 2010;15(4):355-63.

6. Şilte Karamanlioğlu D, Aktas I, Ozkan FU, Kaysin M, Girgin N. Effectiveness of ultrasound treatment applied with exercise therapy on patients with ankylosing spondylitis: a double-blind, randomized, placebo-controlled trial. Rheumatol Int. mayo de 2016;36(5):653-61.

7. World Medical Association. World Medical Association Declaration of Helsinki: Ethical Principles for Medical Research Involving Human Subjects. JAMA. 27 de noviembre de 2013;310(20):2191-4.
