# Supplementary material for: Efficacy of myofascial induction compared with its simulation on joint amplitude in people with axial spondylarthritis: Protocol of a randomized controlled clinical trial
Source: PLoS One. 2023 Oct 5;18(10):e0286885. doi: 10.1371/journal.pone.0286885 (PMC10553292; doi:10.1371/journal.pone.0286885)
Supplement: S2 File — (DOCX) [file pone.0286885.s003.docx]

**TÍTULO DEL ENSAYO CLÍNICO: EFICACIA DE LA INDUCCIÓN MIOFASCIAL COMPARADO CON TERAPIA MANUAL SIMULADA SOBRE LA AMPLITUD ARTICULAR EN PACIENTES CON DIAGNÓSTICO DE ESPONDILOARTRITIS AXIAL**

| **Investigador principal:** | María Alejandra Sánchez Vera, Fisioterapeuta. Profesora Facultad de Enfermería y Rehabilitación. Universidad de La Sabana. |
| --- | --- |
| **Patrocinador:** | Universidad de La Sabana  Campus del Puente del Común, Km. 7, Autopista Norte de Bogotá. Chía, Cundinamarca, Colombia. |

# CONTACTO INVESTIGADOR PRINCIPAL

| **Investigador principal** | María Alejandra Sánchez Vera, Fisioterapeuta, Especialista en Terapia Manual Ortopédica, estudiante Maestría en Epidemiología. Profesora Facultad de Enfermería y Rehabilitación, Universidad de La Sabana. Chía, Colombia. |
| --- | --- |
| **Patrocinador** | Universidad de La Sabana. |
| **Financiadores** | No aplica |
| **Estadístico** | Análisis estadísticos realizados por la investigadora principal |
| **Comité** | No aplica |

# RESUMEN

| **Título del ensayo** | Eficacia de la inducción miofascial comparado con terapia manual simulada sobre la amplitud articular en pacientes con diagnóstico de espondiloartritis axial. |
| --- | --- |
| **Registro del ensayo** | NCT04424589 |
| **Patrocinador** | Universidad de La Sabana |
| **Financiador** | No aplica |
| **Fase clínica** | Ensayo clínico fase III |
| **Diseño del ensayo** | Ensayo clínico aleatorizado paralelo de superioridad, doble ciego, controlado. |
| **Participantes del ensayo** | Personas con diagnóstico de espondiloartritis axial, mayores de edad que asistan al servicio de reumatología de una IPS ambulatoria en la ciudad de Bogotá. |
| **Tamaño de muestra** | 82 personas en total. |
| **Periodo planeado para el ensayo** | La intervención por participante se tiene diseñada para desarrollar 6 sesiones de terapia 2 veces por semana en el transcurso de 3 semanas. Se realizará seguimiento cuatro semanas posterior a la intervención de las variables medidas de forma basal. |

# OBJETIVOS Y MEDIDAS DE RESULTADO

| **OBJETIVO** | **MEDIDA RESULTADO** | **TIEMPO DE MEDICIÓN** |
| --- | --- | --- |
| **Objetivo general:**  Evaluar la eficacia de la inducción miofascial comparado con terapia manual simulada en la amplitud articular sobre el cambio del BASMI en pacientes con diagnóstico de espondiloartritis axial. | **BASMI:** Se analizará el puntaje global de este índice, así como la diferencia de promedios de cada una de las variables que lo componen: (Distancia intermaleolar, distancia trago pared, test de Schober, rotación cervical, test de flexión lateral) | A la semana 1 sesión previa de examen, se realiza la medición basal entre los participantes, a la semana 3 sesión 6 se realiza la medición final y posteriormente se medirá a la cuarta semana terminada la intervención. |
| Describir cambios en términos de calidad de vida, y funcionalidad de la EspaAx entre los grupos a través del análisis de los puntajes de los índices BASFI, ASQoL. | BASFI: Permite la medición del nivel de funcionalidad de los pacientes con EspaAx  ASQoL: Permite la medición del nivel de calidad de vida entre los pacientes con EspaAx. | Medición de estos índices en la semana 1 sesión de examen, medición final en la semana 3 sesión 3 y posteriormente se medirá a la cuarta semana terminada la intervención. |

**IDENTIFICACIÓN DEL PARTICIPANTE**

**Participantes del ensayo**

Se incluirán participantes mayores de edad con diagnóstico de espondiloartritis axial que asistan a una IPS ambulatoria en Bogotá, este diagnóstico debe ser confirmado por reumatólogo y deberá estar consignado en la historia clínica, se incluirán pacientes independientemente del tipo de EspaAx, el nivel de actividad, severidad o tiempo de diagnóstico de la enfermedad.

**Criterios de inclusión**

- Pacientes de ambos sexos mayores de 18 años.
- Pacientes con diagnóstico confirmado por reumatología de EspaAx independientemente del nivel de actividad de la enfermedad.
- Pacientes con capacidad cognitiva para seguimiento de órdenes.
- Que acepte participar en el estudio y firme consentimiento informado.

**Criterios de exclusión:**

- Pacientes que reciban terapia de coagulación oral o parenteral.
- Mujeres embarazadas
- Pacientes con Kinesiofobia.
- Pacientes con fisioterapia previa en los últimos 15 días.
- Presencia de cáncer activo, tratamiento actual en quimio o radioterapia.
- Pacientes que no deseen participar en el estudio

**RECLUTAMIENTO**

Los pacientes que podrán ingresar al estudio son aquellas personas que estén recibiendo algún servicio médico o terapéutico en la IPS de Bogotá. En aras de realizar el proceso de reclutamiento para el estudio, un auxiliar de investigación revisará las historias clínicas de los pacientes que tengan diagnóstico de EspaAx confirmado por reumatólogo, es de esperarse que en este primer tamizaje algunas historias no cumplan con los criterios de inclusión, sin embargo para aquellos pacientes que sean potencialmente elegibles, el auxiliar los contactará vía telefónica y explicará de manera breve la intención y el alcance del estudio, posterior a esta comunicación si el paciente manifiesta de forma verbal su deseo de hacer parte del proceso investigativo, se agendará un espacio de encuentro presencial donde se explique la dinámica del estudio.

Una vez se lleve a cabo el encuentro presencial con el posible participante, en esta sesión se les explicará de forma suficiente y completa el consentimiento informado, manifestando de forma verbal y escrita los potenciales riesgos y ventajas de este proceso terapéutico, así mismo se llevará a cabo el proceso de examen basal con los índices de funcionalidad, movilidad, actividad de la enfermedad y calidad de vida relacionados dentro de la investigación. Con base en esta evaluación se tendrán en cuenta los pacientes que cumplan con los criterios de inclusión.

Siguiendo el debido proceso, el paciente será aleatorizado en uno de los grupos (intervención o control) y asistirá a 6 sesiones de inducción miofascial o terapia manual simulada de acuerdo con el grupo asignado, en la última sesión se realizará la valoración final con los mismos índices que fueron valorados de forma inicial. Los pacientes que recibieron terapia manual simulada recibirán la IMF real una vez finalizado el ENSAYO CLÍNICO, si así lo desean.

**ALEATORIZACIÓN Y OCULTAMIENTO**

En este estudio se utilizará un proceso de aleatorización simple por medio de la asignación de números aleatorios sin repetición realizado por medio del programa Excel. Este proceso se realizará por una persona externa ajena a la investigación y se garantizará la rigurosidad metodológica por medio de un testigo o árbitro externo adicional. Este proceso se llevará a cabo una vez el paciente haya tenido la sesión número uno de examen y como se mencionó anteriormente se realizará por una persona ajena a la investigación. Para ocultar la asignación al azar, se utilizarán y mantendrán sobres opacos, sellados, numerados consecutivamente y guardados por una única persona. Una persona externa preparará los sobres. Se abrirá un sobre en secuencia una vez que el participante haya completado el examen físico basal y las mediciones correspondientes. La asignación se informará al fisioterapeuta tratante por correo electrónico antes de que el participante asista a la IPS para el tratamiento.

**ENMASCARAMIENTO**

Para garantizar el doble enmascaramiento, las personas que realizarán las evaluaciones basales y finales no conocerán las secuencias de aleatorización ni estarán involucradas en la ejecución de los tratamientos del grupo control ni del experimental, los pacientes recibirán visualmente los mismos procedimientos puesto que las técnicas simuladas lucen exactamente iguales entre sí, de este modo ellos también estarán blindados. Los fisioterapeutas que realizarán las intervenciones no pueden ser enmascarados debido a la necesidad de conocer los principios de la técnica y su forma de ejecución para garantizar una correcta aplicación del tratamiento y de la respectiva simulación

**PROCEDIMIENTOS**

1. Lectura de historias clínicas para filtrar pacientes potencialmente candidatos al estudio.
2. Realice una invitación para participar por teléfono, si la respuesta es positiva, programe una reunión presencial.
3. En la reunión presencial, un investigador que no participe en la interpretación y análisis de los datos deberá realizar el proceso de lectura detallada del consentimiento informado. Una vez aceptado, se procederá al examen basal si el paciente cumple con los criterios de selección, a través del formato físico de toma de datos, aplicando los índices: BASMI, BASDAI, BASFI, ASQoL. La forma de aplicación de estos índices se llevará a cabo de la siguiente manera:

### **Aplicación del BASMI:**

El auxiliar de investigación realizará la medición de cada una de las variables del BASMI a partir del siguiente protocolo de implementación descrito y sugerido por Bath Índices:(1–3)

| **MEDIDA** | **POSICIÓN INICIAL** | **MÉTODO** | **RECOMENDACIONES** |
| --- | --- | --- | --- |
| Distancia trago pared | El paciente permanece en bípedo descalzo, con la espalda pegada a la pared, los hombros idealmente nivelados, las escápulas, los glúteos y los talones contra la pared. Los pies deben permanecer paralelos. El examinador debe garantizar la alineación de la cabeza en la posición más neutral (alineación anatómica) posible. | El paciente estira la barbilla lo más posible (retracción). El examinador observa atentamente y se ubica al lado del paciente en la pared para medir la distancia entre el trago de la oreja y la pared, usando una regla rígida. Se realiza dos veces este procedimiento eligiendo la mejor de las mediciones, la media de ambos lados permite obtener el resultado final | El examinador debe asegurarse de que no se produzca extensión cervical, rotación, flexión o flexión lateral. Evitar que la barbilla supere la horizontal. Además, que el paciente mantenga la retracción mientras se realiza la medición derecha e izquierda. |
| Flexión lateral de tronco | El paciente permanece en bípedo descalzo, con la espalda pegada a la pared, los hombros idealmente nivelados, las escápulas, los glúteos y los talones contra la pared. Los pies deben permanecer paralelos y separados en sus bordes externos a unos 30 cm aproximadamente. | Antes de que ocurra cualquier movimiento, el paciente debe mantener los brazos, las muñensayo clínicos y los dedos junto a su cuerpo, con los dedos en extensión y los hombros deprimidos.  El examinador debe medir desde la punta del dedo medio hasta el piso. Con los brazos relajados a los lados, el paciente intentará alcanzar el piso haciendo una flexión lateral y manteniendo la depresión del hombro. El examinador debe volver a medir desde la punta del dedo medio hasta el suelo.  La diferencia entre 2 mediciones representa la cantidad de flexión lateral. Repetir en el otro lado. (1) El resultado es la media de los resultados en ambos lados y se registra el mejor de los intento | El examinador debe asegurarse que el paciente mantenga los brazos, dedos y rodillas rectos y los talones en el piso. Asegurarse, además, de evitar cualquier flexión hacia adelante, extensión o rotación del tronco |
| Flexión lumbar (Schober modificado) | Paciente en bípedo con los pies paralelos a una distancia de 30cms medidos desde los bordes externos. El examinador marca un primer punto en la quinta vértebra lumbar L5 a medio camino de las espinas iliacas postero superiores, un segundo punto está marcado 10 cm por encima de este y un tercer punto 5 cm por debajo del primero para dar una línea general de 15 cm. | El paciente debe realizar una flexión anterior de tronco con las rodillas completamente extendidas. Se mide con una cinta métrica la distancia entre las 2 marcas superior e inferior. Cualquier aumento más allá de 15 cm representa la cantidad de movimiento logrado. Realizar 2 intentos y sacar el promedio de las mediciones. | Al final del movimiento, el examinador podría optar por permitir una ligera flexión de la rodilla para disminuir la influencia de los isquiotibiales. Esto debe ser documentado en el formato de registro inicial de los datos. |
| Rotación cervical | Paciente en posición sedente, pies apoyados sobre el suelo, manteniendo una flexión de rodilla de 90°, el fulcro del goniómetro va en el centro de la cabeza del paciente, el brazo móvil seguirá la nariz y el brazo fijo el acromion del lado hacia donde se produce la rotación. | El paciente realiza una rotación activa cervical hacia la derecha y hacia la izquierda. Se hace el procedimiento dos veces para ambos lados y se registra el mejor de los intentos, el promedio de ambos es el resultado final. | El examinador debe asegurarse que el paciente no realice flexión del cuello o flexión lateral. |
| Distancia intermaleolar | Paciente se encuentra en posición supina con las rodillas en extensión. | El examinador procede a separar lo máximo posible los pies del paciente realizando una abducción de caderas bilateralmente. Se mide la distancia entre los maléolos internos con una cinta métrica. | Evitar que esta resulte una medida absolutamente dolorosa para el paciente, realizar una medición rápida. |

Al final se debe registrar la puntuación otorgada por el paciente para cada una de las variables, así como la puntuación total para el análisis entre grupos. La puntuación se realizará mediante la ESCALA BASMI de 11 puntos, el investigador deberá continuar con la evaluación del BASFI, BASDAI, ASQOL, BASMI.

# INTERVENCIONES EN EL ENSAYO CLÍNICO

## **Intervención grupo experimental**

Esta intervención se encuentra en su totalidad basada en las técnicas de inducción miofascial propuestas por Andrezj Pilat en su libro de terapias miofasciales: Inducción miofascial. Los fisioterapeutas que ejecutaran estos procedimientos se encuentran formados y certificados en esta técnica por el autor en mención. (4)

**Técnica en Jota:**

Una vez identificado y examinada la restricción fascial procederá a fijar con su mano no dominante en sentido opuesto a la dirección de la restricción, realizando una ligera tracción superficial que ayude a mantener tenso el tejido para la maniobra a realizar. Cuando se fija en sentido opuesto a la restricción, el fisioterapeuta con su mano dominante realiza un ascenso del tercer dedo sobre el segundo nivel de la falange distal, el antebrazo estará en pronación, el codo en ligera flexión, los hombros en descenso, luego procederá a realizar la técnica en “J” donde iniciará realizando un deslizamiento lento de en promedio 4cms, con profundidad superficial no dolorosa y sobre el sitio de la restricción miofascial, realizará una rápida supinación en forma de “J”, es probable que se observen respuestas vasomotoras como sudoración y eritema temporal de la piel de la espalda. Por cada uno de los sitios percibidos como adheridos o restringidos se deben realizar en promedio 7 jotas. Esta técnica no necesita ningún lubricante o medio de contacto.

**Liberación de la fascia paravertebral**

La sesión de inducción miofascial continuará con el paciente en posición sedente con la espalda descubierta, los contactos del FT serán los nudillos del segundo, tercer y cuarto dedo, se le pedirá al participante que flexione la cabeza, mientras de deslizan los contactos hasta la segunda vértebra torácica. Se detendrá el movimiento, en ese momento el terapeuta llevará hacia atrás una de sus extremidades inferiores, en este punto continuará la maniobra manteniendo los codos en extensión y apoyándose de su mecánica corporal le pide al participante que se flexione lentamente hacia adelante mientras mantiene las piernas en libera abducción, mientras el FT realiza un deslizamiento caudal de los contactos hasta llegar a las vértebras lumbares altas, en cada sitio donde fuese percibida una restricción de movimiento se detendrá sin aumentar la presión por 7 segundos. El paciente deberá permanecer en esta posición hasta que el FT lentamente le ayude a incorporarse nuevamente a la posición sedente. Repetir 3 veces.

En caso que el paciente al momento de la sesión reporte exacerbación de los síntomas dolorosos por encima de 6/10 según la EAV, se realizará esta misma maniobra pero en posición prona, con la espalda descubierta, el terapeuta manual con la mano externa (no dominante) realiza una fijación hacia craneal a modo de ligera tracción sin generar presión sobre la región cervical baja (C6-C7-T1), con su mano interna o dominante fleja su segundo o tercer dedo y con la articulación interfalángica proximal realiza un deslizamiento longitudinal, con una presión sostenida, lenta y no dolorosa a lo largo del recorrido del músculo paravertebral. En todos los puntos de restricción miofascial percibidos (Varían en relación con el paciente), el fisioterapeuta se detendrá por 7 segundos sin aumentar la presión ejercida o generar dolor. Esta maniobra de deslizamiento la realizará 3 veces por cada erector espinal. Esta técnica no necesita ningún lubricante o medio de contacto adicional.

**Técnica de liberación del cuadrado lumbar:**

El paciente se encontrará en posición de cúbito lateral con flexión de rodilla y cadera de 90°, el FT permanecerá en bípedo frente al paciente a la altura del tronco, la mano craneal fijará las costillas en sentido ascendente y la mano distal con el borde cubital de la mano se ubicará en el espacio entre la cresta iliaca y la última costilla, el FT buscará ganar profundidad en este espacio hasta sentir resistencia, una vez llega a la máxima barrera permitida del tejido realizará un movimiento de flexión y extensión del codo de baja amplitud, rítmico en 15 ciclos en 3 repeticiones.

En un segundo momento, en esa misma posición el paciente dejará caer ligeramente las piernas por fuera de la camilla el FT acompañará esta puesta en tensión del cuadrado lumbar localizando los pulpejos de los dedos dentro de la masa común paravertebral mientras el antebrazo caudal permanecerá entre la cresta iliaca y el trocánter y el antebrazo craneal en la parrilla costal, el FT realizará un deslizamiento transverso con sus dedos sin generar flexión del carpo, realiza 13 ciclos en 3 repeticiones.

Finalizará esta técnica con el paciente decúbito lateral dejará caer ligeramente las piernas por fuera de la camilla, el FT tendrá las manos cruzadas una sobre la cresta iliaca y otra sobre la parrilla costal, iniciará generando ligera tracción sobre la piel llevando a un estado de pretensión el tejido, después con una ligera presión intentará vencer las barreras propias del tejido facilitando el movimiento de la fascia con una presión sostenida, ligera y duradera en el tiempo por 5 minutos.

Una vez finalizada la técnica se retirará lentamente la presión de la zona, y antes se retirar las manos del segmento se mantendrá el contacto manual por 10 segundos, una vez retiradas las manos se permitirá el reposo del paciente en la camilla por 2 minutos aproximados para evitar respuestas vaso vágales. Realizar de forma bilateral las técnicas mencionadas anteriormente.

**Liberación de la fascia sacroilíaca:**

El paciente permanecerá en prono con flexión de rodilla a 90°, el FT permanecerá en bípedo al lado de la extremidad a trabajar, su mano craneal reposará sobre el sacro en dirección de las crestas iliacas, mientras mantiene la rotación interna con la mano caudal de esa extremidad, sostendrá la presión hasta sentir que sobrepasa entre 3 y 6 barreras tisulares, sostener por 4 minutos.

**Técnica de presión sostenida en trapecio superior:**

Para el cuello, se utilizará la técnica de liberación miofascial en trapecio superior, el paciente se encontrará en supino el fisioterapeuta a la altura de la cabecera de la camilla sentado, posicionará sus manos de la siguiente forma: Su mano no dominante estará dándole soporte a la mano dominante con una flexión de 90° a nivel de las articulaciones metacarpofalángicas, el contacto inicial con los músculos trapecio superior y angular del omoplato iniciará de forma suave y progresiva con las punta de los dedos, aumentando la presión y profundidad de la posición de las manos.

El FT sentirá cada una de las barreras tisulares cediendo lentamente ante la presión, en primer lugar, sentirá ligera resistencia de la piel, seguido por la fascia superficial y músculo, hasta que llegará a la profundidad esperada y permitida por los diferentes tejidos, esta posición se mantendrá por 5 minutos. No se realizará ningún tipo de deslizamiento o movimiento accesorio en esta técnica. No necesita ningún tipo de lubricante.

**Técnica de manos cruzadas en fascia toracolumbar:**

La intervención continúa con la técnica de manos cruzadas, en este momento el fisioterapeuta se ubicará a un lado de la camilla, el paciente con la espalda descubierta y en posición prona. El fisioterapeuta sincronizará su respiración con la del paciente, una vez este ha tomado dos inhalaciones profundas, el terapeuta ubicará completamente las palmas de sus manos sobre la espalda del paciente a nivel de la región lumbo sacra con los dedos en ligera abducción, iniciará generando ligera tracción sobre la piel llevando a un estado de pre tensión el tejido, después con una ligera presión intentará vencer las barreras propias del tejido facilitando el movimiento de la fascia con una presión sostenida, ligera y duradera en el tiempo por 5 minutos.

Una vez finalizada la técnica se retirará lentamente la presión de la zona, y antes se retirar las manos del segmento se mantendrá el contacto manual por 10 segundos, una vez retiradas las manos se permitirá el reposo del paciente en la camilla por 2 minutos aproximados para evitar respuestas vaso vágales como mareos, síncopes, entre otros.

## **Intervención del grupo control**

La intervención de simulación en el grupo control sugerirá la modificación de parámetros básicos que no alteren la demostración visual de la técnica. El fisioterapeuta asignado a este grupo omitirá los principios básicos de la inducción miofascial: tracción, presión sostenida y profundidad del tejido. Se llevarán a cabo todas las estrategias propuestas para el grupo experimental.

**EVENTOS ADVERSOS**

Los eventos adversos se evaluarán en el formato designado para tal fin incluido en el apéndice del protocolo. En términos generales, el formato de reporte incluye información que será diligenciada por el paciente y otra que debe ser organizada por el investigador principal. los EA incluidos serán aquellos que ocurrieron durante el tratamiento o en las 24 horas posteriores al mismo. El cuestionario de eventos adversos contiene de forma general las preguntas relacionadas a si el paciente había experimentado esa sensación antes durante los procedimientos de intervención, así mismo se enfocan en indagar cuántas horas duró el evento y en qué medida el EA habría generado malestar físico en el participante, esto será medido a través de una calificación numérica como la escala visual análoga (EVA) de 11 puntos de 0 a 10 (0 = no lo molesta en absoluto y 10 = lo molestó de la peor manera posible).(5)

Esta clasificación será realizada por el investigador principal a partir del formulario diseñado para tal fin. Se dejará al juicio clínico del investigador decidir si un EA es o no de gravedad suficiente para requerir la retirada del tratamiento del participante. Un participante también puede retirarse voluntariamente del tratamiento debido a lo que él o ella percibe como un EA intolerable. Si ocurre cualquiera de estos, el participante debe someterse a una evaluación de finalización del ensayo y recibir la atención adecuada bajo supervisión médica o fisioterapéutica hasta que los síntomas desaparezcan o la condición se estabilice.

## **Plan de análisis de datos**

##### Se construirá un formulario de recolección físico que incluya las variables a medir para un registro físico, posterior a esto se creará un formulario virtual en RedCap. Se verificará el adecuado registro a partir de la doble digitación, en caso de datos faltantes se contactará a la fuente primaria (paciente) y se realizaran las correcciones.

##### Se realizará un análisis estadístico de normalidad para las variables cuantitativas con la prueba de Shapiro Wilk o Kolmogorov Smirnoff acorde a los datos, seguido a esto se realizará un análisis descriptivo, para las variables continuas análisis de medidas de tendencia central si presentan distribución normal y mediana o dispersión si no presentan distribución normal. Para las variables categóricas se calcularán las proporciones y frecuencias.

##### Las diferencias entre los grupos para la medición de los objetivos primarios y secundarios se analizarán con la prueba t de Student para las variables continuas que presenten distribución normal y con la prueba de Mann Whitney Wilcoxon para las variables con distribución no normal. Para las variables cualitativas se evaluarán con la prueba de independencia de Ji-cuadrado o la prueba exacta de Fisher según corresponda. Para todas las pruebas se aceptará un nivel de significancia de (p < 0.05).

**PROCEDIMIENTOS ÉTICOS:**

Este ensayo clínico desde su planteamiento y formulación se adhiere a los estándares nacionales e internacionales de reglamentación ética, de manera particular a la declaración de Helsinki promulgada por la asociación médica mundial como una propuesta de principios éticos para la investigación médica en seres humano. Este estudio, busca realizar y evaluar la eficacia de un procedimiento que tiene potencial valor terapéutico que no afecta ni pone en riesgo la integridad física o la salud de los participantes. Tal como lo cita esta declaración la importancia del objetivo perseguido en el estudio es mayor que el riesgo y los costos para el participante. Así mismo se reitera que la participación de la persona en este ENSAYO CLÍNICO es completamente voluntaria y reglamentada a través de un consentimiento informado que deberá ser aprobado por un comité de ética, así como el protocolo de investigación. Es importante mencionar que en concordancia con la citada declaración se garantizara permanentemente la privacidad e intimidad de los participantes respecto al registro de los datos y la divulgación de estos

## **DETERMINACIÓN DEL TAMAÑO MUESTRAL Y MUESTREO**

Para calcular el tamaño muestral del ensayo clínico se toma como referencia el artículo de Silte y cols(6), debido a que no existen puntos de corte en Colombia o Latinoamérica claramente establecidos que diferencien puntajes de normalidad del BASMI entre población con y sin presencia de EspaAx, adicionalmente no hay disponibles a la fecha ensayo clínico realizados en esta población tomando como intervención primaria o experimental técnicas desde la terapia manual. Dado lo anterior, se hace necesario citar un referente teórico para realizar las estimaciones del cálculo de tamaño muestral. Se eligió un estudio que cumpliese con el análisis de cada una de las variables del BASMI y que desarrollara una comparación entre grupos de dos técnicas y estrategias desde fisioterapia.

Con base en lo anterior, se tiene como referencia el estudio en cuestión tomando los promedios del grupo control y experimental post intervención con sus respectivas desviaciones estándar por cada una de las variables del índice BASMI, seguido a esto se estima y construye entre los investigadores del estudio los deltas esperados que se consideraron relevantes en la práctica clínica habitual en consulta de fisioterapia y reumatología.

Se realiza el cálculo utilizando en el programa R Studio última versión 3.5.3 descargando la librería EpiR, junto con el paquete de ensayos clínicos de superioridad denominado Epi.supc, una vez finalizado este proceso, se procede a correr el siguiente código:

epi.supc (treat = 1, control = 1, sd = 1, delta = 1, n = NA, r = 1, power = 0.80, alpha =0.05)

Siendo treat el promedio esperado en el grupo experimental, control el promedio esperado en el grupo control, sd la desviación estándar, delta el cambio mínimo que representa importancia clínica, r la razón entre grupos. Se estimó para la realización de todos los cálculos un alfa de 0.05% y una potencia de 0.80. Este cálculo se realizó para cada una de las variables del BASMI (Flexión anterior de tronco, flexión lateral de tronco, rotación cervical, distancia intermaleolar, distancia trago pared) y se seleccionó el mayor tamaño muestral calculado, este fue de 33 pacientes por brazo. Tomando en cuenta un porcentaje de pérdida de los participantes del 20% el total de pacientes es de 82 en total (42 por brazo). Se utilizará un muestro no probabilístico por intención.

**PROCEDIMIENTOS ÉTICOS**

Este ECA desde su planteamiento y formulación se adhiere a los estándares nacionales e internacionales de reglamentación ética, de manera particular a la declaración de Helsinki promulgada por la asociación médica mundial como una propuesta de principios éticos para la investigación médica en seres humanos. Este estudio, busca realizar y evaluar la eficacia de un procedimiento que tiene potencial valor terapéutico que no afecta ni pone en riesgo la integridad física o la salud de los participantes. Tal como lo cita esta declaración la importancia del objetivo perseguido en el estudio es mayor que el riesgo y los costos para el participante. Así mismo se reitera que la participación de la persona en este ECA es completamente voluntaria y reglamentada a través de un consentimiento informado que deberá ser aprobado por un comité de ética, así como el protocolo de investigación. Es importante mencionar que en concordancia con la citada declaración se garantizara permanentemente la privacidad e intimidad de los participantes respecto al registro de los datos y la divulgación de estos(7)

**REFERENCIAS**

1. Thomas A, Martindale J. An updated synopsis of the Bath Indices – outcome measures for use with Ankylosing Spondylitis patients and their broader application. :16.

2. Castro Villegas M del C, Batlle Gualda E. Metrología en espondiloartritis. Reumatol Clin. 1 de marzo de 2010;6:11-7.

3. Calin A, Jones SD, Garrett SL, Kennedy LG. Bath Ankylosing Spondylitis Functional Index. Br J Rheumatol. agosto de 1995;34(8):793-4.

4. Pilat A, Gonzalez Nieto JL, Batuecas Suárez A. Terapias miofaciales : inducción miofascial. Aséctos teóricos y aplicaciones clínicas / Andrzej Pilat ; revisión técnica Alicia Batuecas Suárez, José Luis González Nieto. [Internet]. 2003 [citado 27 de marzo de 2019]. Disponible en: http://unisabana22.gsl.com.mx:80/F?func=service&doc_library=CNA01&local_base=CNA01&doc_number=000065058&sequence=000001&line_number=0001&func_code=DB_RECORDS&service_type=MEDIA

5. Carnes D, Mars TS, Mullinger B, Froud R, Underwood M. Adverse events and manual therapy: a systematic review. Man Ther. agosto de 2010;15(4):355-63.

6. Şilte Karamanlioğlu D, Aktas I, Ozkan FU, Kaysin M, Girgin N. Effectiveness of ultrasound treatment applied with exercise therapy on patients with ankylosing spondylitis: a double-blind, randomized, placebo-controlled trial. Rheumatol Int. mayo de 2016;36(5):653-61.

7. World Medical Association. World Medical Association Declaration of Helsinki: Ethical Principles for Medical Research Involving Human Subjects. JAMA. 27 de noviembre de 2013;310(20):2191-4.
